# Comprehensive comparison of three techniques for the treatment of adjacent segment degeneration after lumbar fusion

**DOI:** 10.3389/fsurg.2023.1096483

**Published:** 2023-03-30

**Authors:** Tao Li, Hang He, Tonghui Zhang, Xugui Li, Wei Xie, Biwang Huang, Feng Xu, Chengjie Xiong

**Affiliations:** ^1^ Department of Orthopaedics, Affiliated Hospital of Wuhan Sports University, Wuhan, China; ^2^ Department of Orthopaedics, General Hospital of Central Theater Command of PLA, Wuhan, China

**Keywords:** PTED, endoscopic surgery, cortical bone trajectory screw, adjacent segment degeneration, minimally invasive spine surgery

## Abstract

**Purpose:**

Adjacent segment degeneration (ASD) following lumbar fusion is technically challenging for spine surgeons. Posterolateral open fusion surgery with pedicle screw fixation is an effective way to treat symptomatic ASD with favorable clinical outcomes; however, it is associated with an increased morbidity rate. Therefore, minimally invasive spine surgery is advocated. This study was designed to compare clinical outcomes among patients with symptomatic ASD who underwent percutaneous transforaminal endoscopic discectomy (PTED) with the transforaminal approach, posterior lumbar interbody fusion (PLIF) with cortical bone trajectory screw fixation (CBT-PLIF), and PLIF with traditional trajectory screw fixation (TT-PLIF).

**Methods:**

A retrospective study was conductedon 46 patients (26 men and 20 women; average age 60.8 ± 6.78 years) with symptomatic ASD. The patients were treated with three approaches. The operation time, incision length, time to return to work, complications, and the like were compared among three groups. Intervertebral disc (IVD) space height, angular motion, and vertebral slippage were obtained to assess spine biomechanical stability following surgery. The visual analog scale (VAS) score and Oswestry disability index were evaluated at preoperation and 1-week, 3-month, and the latest follow-ups. Clinical global outcomes were also estimated using modified MacNab criteria.

**Results:**

The operation time, incision length, intraoperative blood loss, and time to return to work for the PTED group were significantly decreased compared with those for the other two groups (*P *< 0.05). The radiological indicators in the CBT-PLIF group and TT-PLIF group had better biomechanical stability compared with those in the PTED groups at the latest follow-up (*P *< 0.05). The back pain VAS score in the CBT-PLIF group was significantly decreased compared with those in the other two groups at the latest follow-up (*P *< 0.05). The good-to-excellent rate was 82.35% in the PTED group, 88.89% in the CBT-PLIF group, and 85.00% in the TT-PLIF group. No serious complications were encountered. Two patients experienced dysesthesia in the PTED group; screw malposition was found in one patient in the CBT-PLIF group. One case with a dural matter tear was observed in the TT-PLIF group.

**Conclusion:**

All three approaches can treat patients with symptomatic ASD efficiently and safely. Functional recovery was more accelerated in the PTED group compared with the other approaches in the short term; CBT-PLIF and TT-PLIF can provide superior biomechanical stability to the lumbosacral spine following decompression compared with PTED; however, compared with TT-PLIF, CBT-PLIF can significantly reduce back pain caused by iatrogenic muscle injury and improve functional recovery. Therefore, superior clinical outcomes were achieved in the CBT-PLIF group compared with the PTED and TT-PLIF groups in the long term.

## 1. Introduction

Adjacent segment degeneration (ASD) is one of the common complications following lumbar spine fusion. It is defined as pathological changes at levels adjacent to fusion segments on radiographic images. It is widely recognized that ASD is caused by the increased range of motion (ROM) at segments adjacent to fused segments (1). The incidence of symptomatic ASD is reported to be as high as 30% (2). Posterior lumbar revision surgery for patients with symptomatic ASD is technically challenging. In the past few decades, posterior lumbar interbody fusion (PLIF) was regarded as a “golden standard” for the treatment of symptomatic ASD (3). These conventional open operations can achieve favorable outcomes in a few cases; however, they are associated with significant morbidities, especially in the elderly (4–6).

With the development of minimally invasive spine surgery (MISS), several alternative solutions are considered and advised in decision-making for symptomatic ASD revision surgery. Percutaneous transforaminal endoscopic discectomy (PTED) is one of the most widely used MISS operations to treat patients with symptomatic ASD (7). PTED has been reported to achieve equivalent or improved clinical outcomes in treating symptomatic ASD. Compared with conventional open operations, it can significantly reduce operation-related trauma, blood loss, and hospital stay (8). This approach is especially valuable for the elderly with chronic diseases who cannot tolerate open operations. Moreover, PTED can also avoid the scar caused by the previous operation and decrease the risks of nerve root injury. However, several studies have reported that the incidence of symptomatic disc herniation at the index segment following this PTED was as high as 3.6% (9). Therefore, other MISS operations should be considered in addition to PTED.

Cortical bone trajectory screw (CBT) is a long-standing technique that was first described by Santoni et al. in 2009 (10). Minimally invasive interbody fusion combined with CBT can significantly reduce paraspinal muscle damage compared with conventional TLIF/PLIF (7, 11). However, studies have demonstrated that PTED and TT-PLIF were applied to treat symptomatic ASD with favorable clinical results. However, few studies have evaluated minimally invasive interbody fusion combined with CBT to treat symptomatic ASD. Therefore, this retrospective study was conducted to testify to the efficacy and safety of CBT-PLIF in managing patients with symptomatic ASD, and either PTED or TT-PLIF was used as referenced standard.

## 2. Materials and methods

### 2.1. Patient demographics

Between August 2015 and August 2018, 46 patients who met the inclusion were enrolled. Seventeen patients were treated with PTED, nine were treated with CBT-PLIF, and twenty were treated with TLIF. All procedures were approved by the affiliated Hospital of Wuhan Sports University Research Ethics Committee and in accordance with the Declaration of Helsinki. Written informed consent was obtained from each participant. The indications are defined as follows: (1) previously lumbar pedicle screw fixation and/or interbody fusion; (2) recurrent sciatica subsequent to a painless period for at least half a year; (3) lumbar disc herniation (LDH)/spinal stenosis at segments adjacent to the fused segments; and (4) conservation treatments (medications or physical therapy) failure for more than 6 weeks (7, 12).

The contraindications are as follows: (1) dynamic spinal instability was observed; (2) degenerative spondylolisthesis more than Meyerding grade I (13); (3) combined with peripheral nerve disease; and (4) patients with severe cardiopulmonary diseases were unable to tolerate the operation.

### 2.2. Surgical approach

This is a retrospective study in which surgical procedures are selected without any difference after admission. The choice of surgical procedure is determined by the requirements of patients and the surgical expertise of the operators.

### 2.3. PTED approach

**Figure 1:**
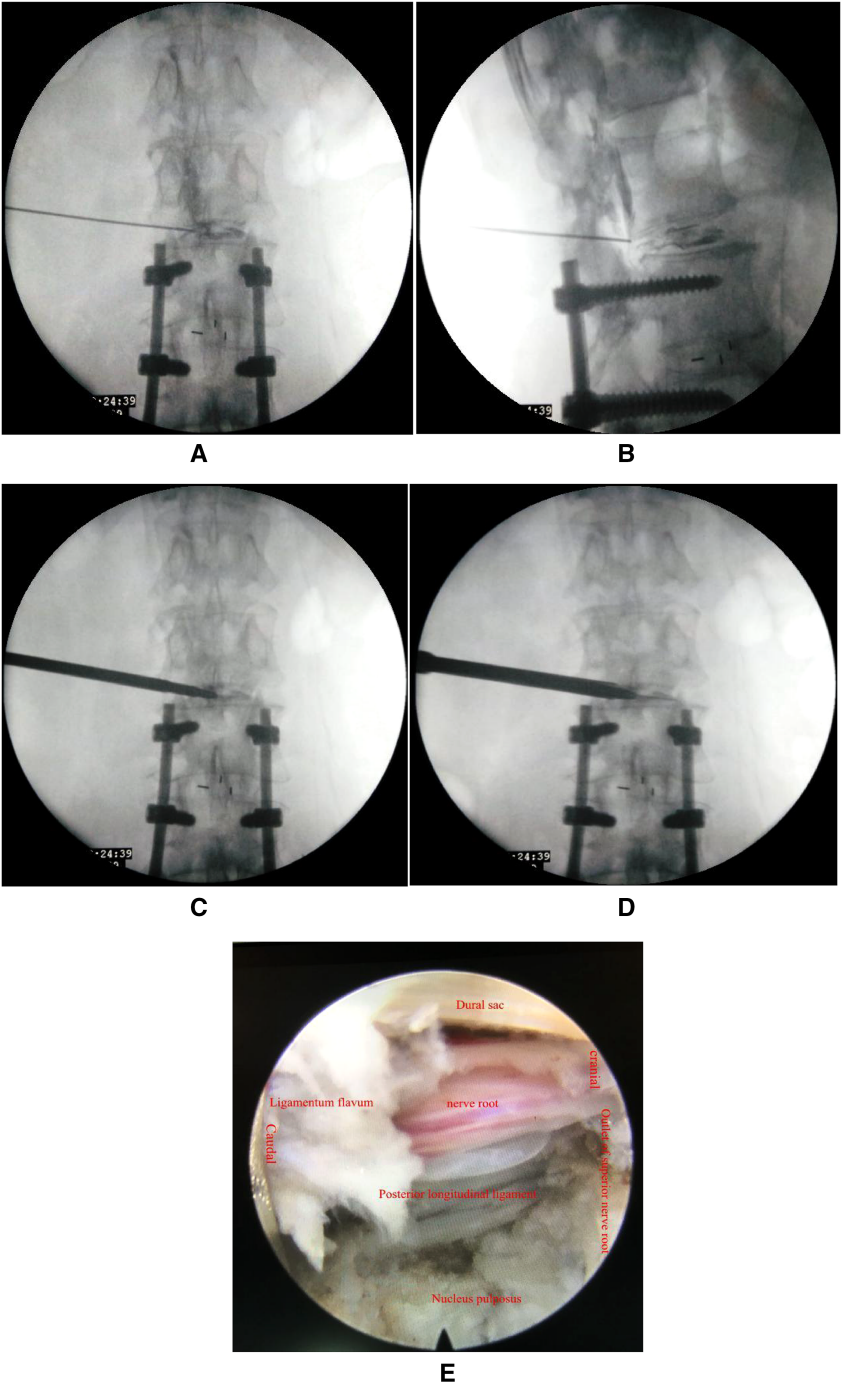
(**A,B**) The spine needle was inserted and the target point was approached under fluoroscopic guidance; (**C**) sequential dilators were introduced through the guide needle; (**D**) a bevel-ended working cannula was placed appropriately; and (**E**) satisfactory decompression was determined by restoration of dural pulsation.

The operation was performed in the prone position. The target intervertebral disc (IVD) space was determined by C-arm fluoroscopy. The entry point was approximately 10–14 cm lateral to the midline. Following local anesthesia, an 18-gauge spinal needle was inserted and the target point was approached with an appropriate trajectory under fluoroscopic guidance. Sometimes, it is necessary to adjust the trajectory of the spinal needle depending on the position of the transverse process or ilium (especially at the L5/S1 level). The remaining steps are as follows: (1) a guide wire was inserted into the spinal needle, and the spinal needle was removed; (2) a skin incision was made at the entry point; (3) sequential dilators were then passed over the guide needle (Figures 1A,B); (4) a four-graded reamer was applied in sequence to enlarge the intervertebral foramen under fluoroscopic guidance (Figure 1C); (5) a bevel-ended working cannula was introduced over the dilator and positioned appropriately (Figure 1D); (6) an endoscopic system assembled with an eccentrically placed 2.7-mm working channel and two irrigation channels was inserted through the working cannula; (7) the herniated disc was removed using endoscopic instruments under endoscopy; the extent of decompression was confirmed by the restoration of dural pulsation (Figure 1E); and (8) after decompression, annuloplasty was conducted by a bipolar radiofrequency, and the endoscopic system was withdrawn. The incision was sutured and covered by a sterile dressing.

Minimally invasive interbody fusion combined with the CBT approach: Following general anesthesia, an operation was performed in the prone position. A midline skin incision was made at the target level. Erector spinal muscles were stripped from spinal processes and lamina bilaterally for the exposure of the entry point of CBT screws. The area of the exposed lamina was usually smaller than that of TT-PLIF. The capsule of the facet joint was kept intact. The entry sites and trajectories of CBT screws differed from pedicle screw implantation in TLIF. The entry point of CBT screws was supposed to be the junction of the center of the superior articular process and 1 mm inferior to the inferior border of the transverse process. Generally, the caudal tilt angle was 25° and the medial tilt angle was 10°, adjusted according to the individual anatomical structure based on the preoperative CT scanning. CBT screws were then implanted under fluoroscopic guidance. The interbody fusion process was consistent with TLIF. The incision was sutured and covered by a sterile dressing.

The TT-PLIF approach has been described in a previous publication (14).

All patients were administered antibiotics routinely postoperatively for 48 h. Patients were encouraged to perform physical activities under the protection of an adjustable brace. However, excessive and heavy activities were prohibited for the next 12 weeks following surgery.

### 2.4. Clinical assessment

The preoperative demographic data included age, gender, duration of symptoms, operation level, and follow-up intervals. The intraoperative demographic data includied operation time, incision length, and blood loss. The postoperative demographic data included time point of postoperative ambulation, length of hospital stay, and total cost.

The functional recovery was assessed by the visual analog scale (VAS) score for back and leg pain, Oswestry disability index (ODI) preoperatively, and at each follow-up (1-week, 3-month, and the latest follow-up) at our orthopedic outpatient department. Modified MacNab criteria (15) were also applied to evaluate global outcomes at the latest follow-up. Sometimes, follow-ups were obtained by telephone (30%) or WeChat app (70%).

### 2.5. Radiographic evaluation

**Figure 2:**
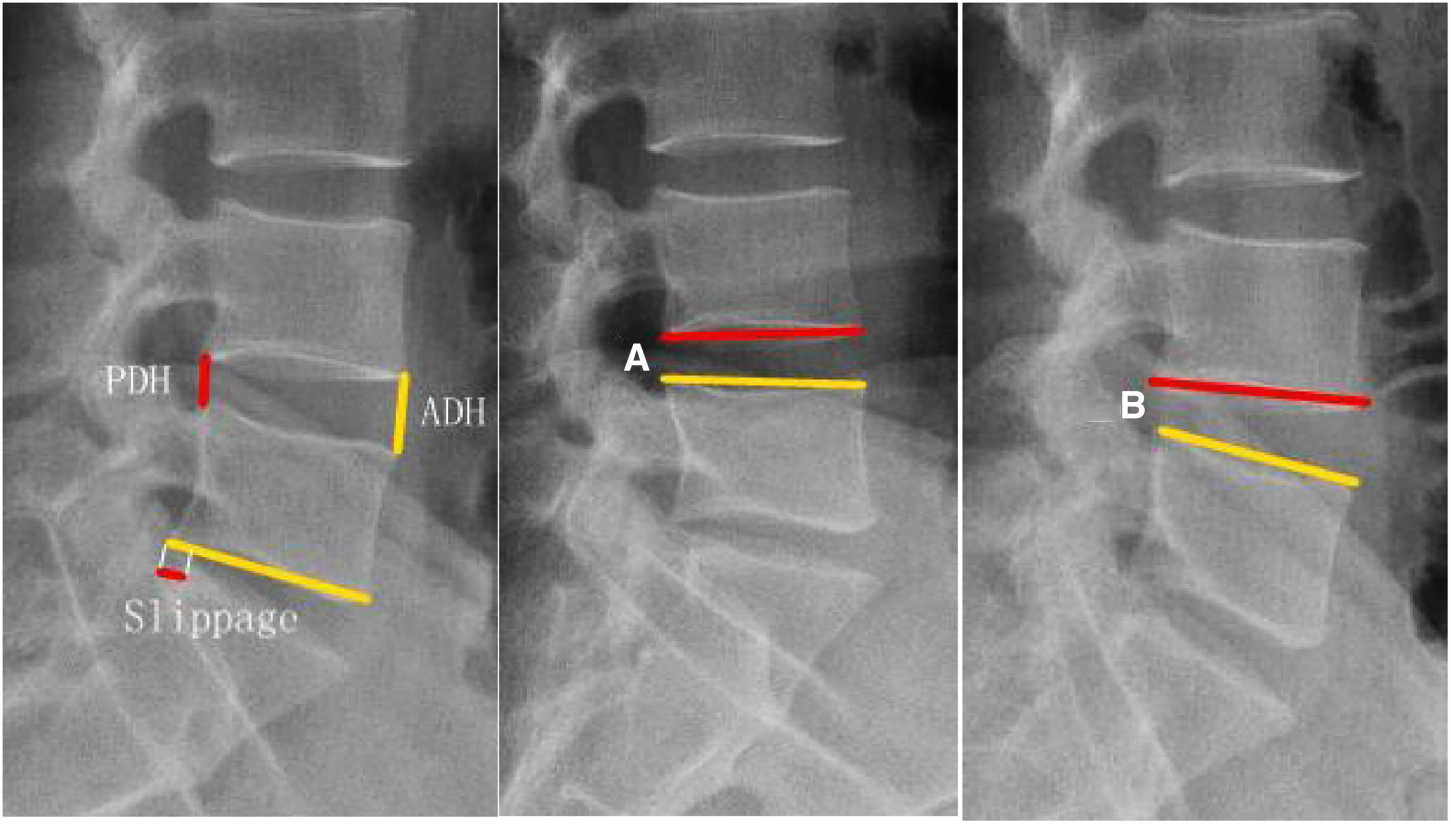
Measurement of radiographic parameters. The IVD space height, posterior disc height (PDH), and anterior disc height (ADH) were obtained and compared. IVD space height = (PDH + ADH)/2; angular motion = Cobb A-Cobb B; translation motion = the length of slippage.

Radiological images were obtained and compared preoperatively and at each follow-up (1-week, 3-month, and the latest follow-up) at our orthopedic outpatient department. Radiographic parameters, including IVD space height, angular motion, and translation motion, were obtained and applied to evaluate spinal stability following the operation. The IVD space height was the distance from the midpoint of the upper endplate to the lower endplate. The angular motion was assessed using plain radiographs between adjacent endplates in flexion and extension. The translation motion was assessed on plain radiographs for one vertebral body on another from the sagittal plane (Figure 2). Dynamic instability was confirmed when translation motion was greater than 3 mm or angular motion was greater than 10° (16, 17).

### 2.6. Statistical analysis

All data were collected by two professional doctors, and a trained team of doctors reviewed the data. The accuracy of the radiological images was evaluated by the coefficient of agreement (kappa), and a kappa value of >0.8 was considered a good agreement (18). Statistical analysis was performed using SPSS version 19.0 software (SPSS, Inc. Chicago, IL, USA). Significant differences between intragroup comparisons were used in the paired sample *t*-test. Significant differences among the three groups were used in the one-way analysis of variance and the SKN-q test. The *χ*^2^ test was used to compare the data between groups, and Fisher's exact test was used when the number was less than five. The grade data were tested using the Kruskal–Wallis rank-sum test. Enumeration data were expressed as rate (%), and measurement data were expressed as mean ± standard deviation. *P* < 0.05 was considered a significant difference.

## 3. Results

### 3.1. Comparison of demographic characteristics

A total of 46 participants (PTED, 17 cases; CBT-PLIF, 9 cases; TT-PLIF, 20 cases) were enrolled. The demographic characteristics (age, gender, duration of symptoms, operative level) are presented and compared in Table 1. No significant differences were found between the three groups regarding these demographic characteristics (*P > *0.05).

**Table 1:** Comparison of demographic characteristics among three groups

Characteristics	PTED	CBT-PLIF	TT-PLIF	*P*-value
Age (years)	59.01 ± 8.47	61.78 ± 5.56	61.85 ± 5.58	0.406
Male [*n* (%)]	11 (64.71%)	6 (66.67%)	9 (45.00%)	0.383
Duration of symptoms (months)	9.64 ± 3.95	9.67 ± 3.71	9.40 ± 3.12	0.972
**Operation level involved**				
L3/4	3	2	3	0.879
L4/5	7	3	9	
L5/S1	6	4	8	
L2/3 and L5/S1	1	0	0	
**Fusion level**				
One level	7	3	9	0.840
Multiple levels	10	6	11	
ASD superior to the fused segment	11	6	13	0.995
ASD inferior to the fused segment	6	3	7	
Follow-up (years)	5.05 ± 0.76	5.18 ± 0.92	4.98 ± 0.52	0.778

### 3.2. Comparison of radiological indicators

**Figure 3:**
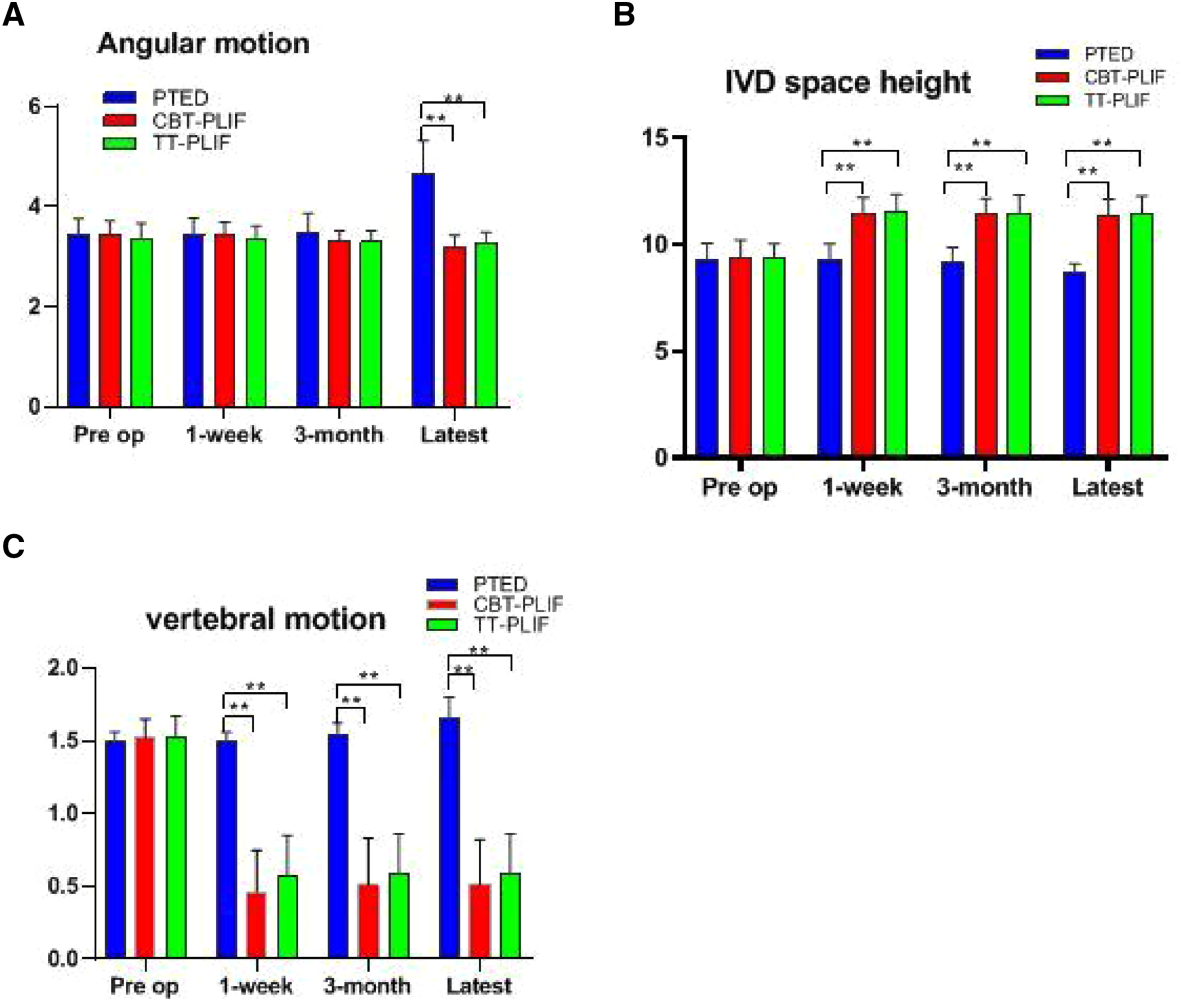
Comparison of radiological indicators among the three groups. Comparison of the (**A**) angular motion, (**B**) IVD space height, and (**C**) vertebral motion at different time points. **P < 0.01, PTED group vs. CBT group, PTED group vs. TT-PLIF group.

No significant differences were found among the three groups regarding the preoperative radiological indicators such as IVD space height, angular motion, and translation motion. The average IVD space height was significantly reduced from 9.29 ± 0.76 to 8.70 ± 0.40 mm in the PTED group, increased from 9.37 ± 0.84 to 11.35 ± 0.78 in the CBT-PLIF group, and increased from 9.36 ± 0.67 to 11.40 ± 0.87 in the TT-PLIF group at the latest follow-up (*P *< 0.05). The average angular motion was significantly increased from 3.43 ± 0.33 to 4.65 ± 0.67 degrees in the PTED group, significantly reduced from 3.44 ± 0.28 to 3.19 ± 0.24 degrees in the CBT-PLIF group, and significantly reduced from 3.37 ± 0.30 to 3.28 ± 0.21 degree in the TT-PLIF group at the latest follow-up (*P *< 0.05). The average translation motion was significantly magnified from 1.49 ± 0.07 to 1.65 ± 0.15 mm in the PTED group. The average translation motion was increased from 1.49 ± 0.07 to 1.65 ± 0.15 in the PTED group, significantly reduced from 1.52 ± 0.13 to 0.51 ± 0.31 in the CBT-PLIF group, and significantly reduced from 1.53 ± 0.14 to 0.58 ± 0.28 in the TT-PLIF group at the latest follow-up (*P *< 0.05). Therefore, the CBT-PLIF group and TT-PLIF group had better biomechanical stability than the PETD group at the latest follow-up (Table 2, Figure 3).

**Table 2:** Comparison of radiological indicators among the three groups.

Follow-up	PTED	CBT-PLIF	TT-PLIF	*P*-value
**IVD space height (mm)**				
Preoperation	9.29 ± 0.76	9.37 ± 0.84	9.36 ± 0.67	0.948
1 week after operation	9.28 ± 0.75	11.44 ± 0.76	11.4 ± 0.87	<0.001
3 months after operation	9.21 ± 0.66	11.40 ± 0.76	11.4 ± 0.88	<0.001
The latest follow-up	8.70 ± 0.40	11.35 ± 0.78	11.4 ± 0.87	<0.001
**Angular motion (degree)**				
Preoperation	3.43 ± 0.33	3.44 ± 0.28	3.37 ± 0.30	0.785
1 week after operation	3.44 ± 0.33	3.42 ± 0.27	3.36 ± 0.25	0.685
3 months after operation	3.49 ± 0.38	3.31 ± 0.21	3.30 ± 0.22	0.118
The latest follow-up	4.65 ± 0.67	3.19 ± 0.24	3.28 ± 0.21	<0.001
**Translation motion (mm)**				
Preoperation	1.49 ± 0.07	1.52 ± 0.13	1.53 ± 0.14	0.576
1 week after operation	1.50 ± 0.06	0.46 ± 0.29	0.57 ± 0.28	<0.001
3 months after operation	1.54 ± 0.08	0.51 ± 0.32	0.58 ± 0.28	<0.001
The latest follow-up	1.65 ± 0.15	0.51 ± 0.31	0.58 ± 0.28	<0.001

### 3.3. Comparison of surgery-related indicators

The operation duration was significantly reduced in the PTED group compared with that in the CBT-PLIF group and the TT-PLIF group (73.06 ± 14.07 vs. 169.44 ± 12.51 vs. 226.50 ± 27.01 min, *P *< 0.05); especially the operation time of ASD superior to fused segments was significantly longer than that below inferior to fused segments in the PTED group (80.91 ± 10.52 vs. 58.67 ± 5.28 min, *P *< 0.05). Therefore, the incision length, intraoperative blood loss, and the time to return to work were significantly reduced in the PTED group compared with those in the CBT-PLIF group and the TT-PLIF group (Table 3).

**Table 3:** Comparison of surgery-related indicators.

Indicators	PTED	CBT-PLIF	TT-PLIF	*P*-value
Operation time (min)	73.06 ± 14.07	169.44 ± 12.51	226.50 ± 27.01	<0.001
Operation time of ASD superior to the fused segment	80.91 ± 10.52	166.17 ± 11.29	226.77 ± 21.46	<0.001
Operation time of ASD below to fused segment	58.67 ± 5.28	176.00 ± 14.53	226.00 ± 37.26	<0.001
Incision length (mm)	8.82 ± 1.47	72.11 ± 4.51	100.40 ± 2.54	<0.001
Intraoperative blood loss (mL)	13.71 ± 3.96	465.33 ± 91.40	759.95 ± 118.69	<0.001
Time to return to work (months)	1.14 ± 0.23	3.21 ± 0.47	3.68 ± 0.58	<0.001

### 3.4. Comparison of clinical and functional outcomes

**Figure 4:**
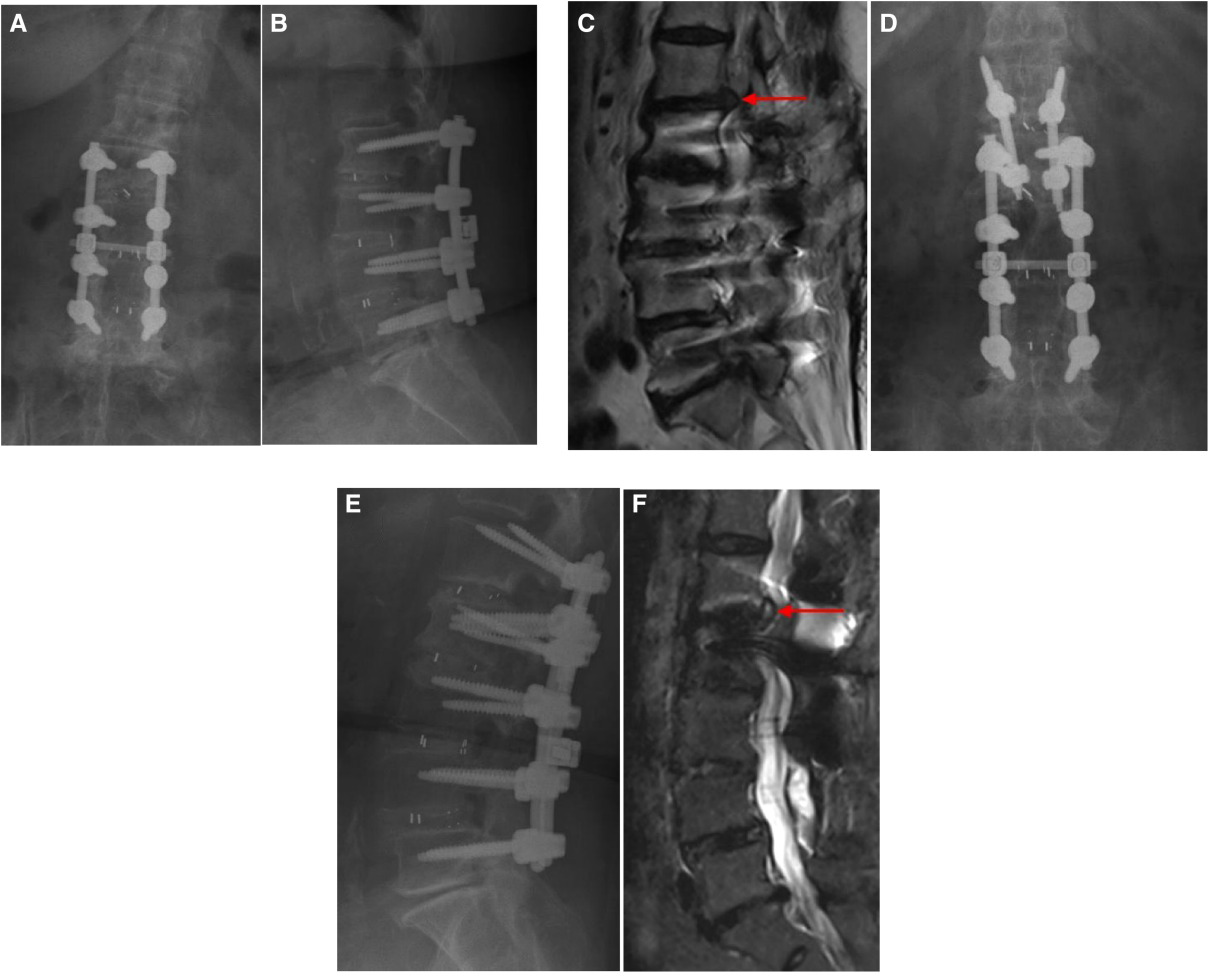
(**A, B**) A patient underwent lumbar 2/3 and L3/4 and lumbar 4/5 TLIF combined with pedicle screw fixation 4 years ago, and numbness and pain recurred in his left thigh 1 year ago. (**C**) MRI of the lumbar spine has shown that lumbar disc herniation was observed at the superior segment adjacent to the fusion segment (L1/2 level), as demonstrated by the red arrows. (**D, E**) Minimally invasive interbody fusion combined with CBT screws fixation was performed at L1/2. (**F**) MRI has demonstrated that the compressed nerve roots were decompressed, as demonstrated by the red arrows. TLIF, transforaminal lumbar interbody fusion; MRI, magnetic resonance imaging; CBT, cortical bone trajectory screw; L1/2, interverbal disc space between lumbar segments 1 and 2.

The average back/leg pain VAS score improved in all three groups following the operation. The average VAS score for leg pain was significantly reduced immediately from 7.65 ± 0.70 to 2.47 ± 0.51 in the PTED group, from 7.78 ± 0.67 to 2.22 ± 0.67 in the CBT-PLIF group, and from 7.80 ± 0.62 to 2.40 ± 0.75 in the TT-PLIF group at 1 week. The average VAS of the back pain was significantly reduced immediately from 5.82 ± 1.42 to 2.47 ± 0.80 in the PTED group, from 6.11 ± 1.05 to 3.44 ± 1.01 in the CBT-PLIF group, and from 5.85 ± 1.23 to 3.70 ± 1.08 in the TT-PLIF group at 1 week. The average ODI score also improved following the operation. The average ODI score was significantly reduced immediately from 62.41 ± 5.28 to 27.35 ± 3.89 in the PTED group, from 63.67 ± 4.77 to 31.67 ± 2.45 in the CBT-PLIF group, and from 62.00 ± 4.81 to 32.45 ± 2.16 in the TT-PLIF group at 1 week (Table 4, Figure 4).

**Table 4:** Comparison of VAS scores for back and leg pain and ODI scores.

Follow-up	PTED	CBT-PLIF	TT-PLIF	*P*-value
**VAS of leg**				
Preoperation	7.65 ± 0.70	7.78 ± 0.67	7.80 ± 0.62	0.774
1 week after operation	2.47 ± 0.51	2.22 ± 0.67	2.40 ± 0.75	0.652
3 months after operation	1.94 ± 0.43	1.89 ± 0.60	1.90 ± 0.55	0.963
The latest follow-up	1.53 ± 0.62	1.44 ± 0.73	1.45 ± 0.69	0.921
**VAS of back**				
Preoperation	5.82 ± 1.42	6.11 ± 1.05	5.85 ± 1.23	0.845
1 week after operation	2.47 ± 0.80	3.44 ± 1.01	3.70 ± 1.08	0.001
3 months after operation	1.88 ± 0.60	2.00 ± 0.50	2.10 ± 0.64	0.545
The latest follow-up	2.35 ± 0.61	1.67 ± 0.50	1.85 ± 0.49	0.005
**ODI**				
Preoperation	62.41 ± 5.28	63.67 ± 4.77	62.0 ± 4.81	0.706
1 week after operation	27.35 ± 3.89	31.67 ± 2.45	32.4 ± 2.16	<0.001
3 months after operation	24.41 ± 2.40	25.67 ± 1.32	25.9 ± 1.85	0.067
The latest follow-up	20.59 ± 1.73	20.56 ± 1.88	20.6 ± 1.79	0.998

**Figure 5:**
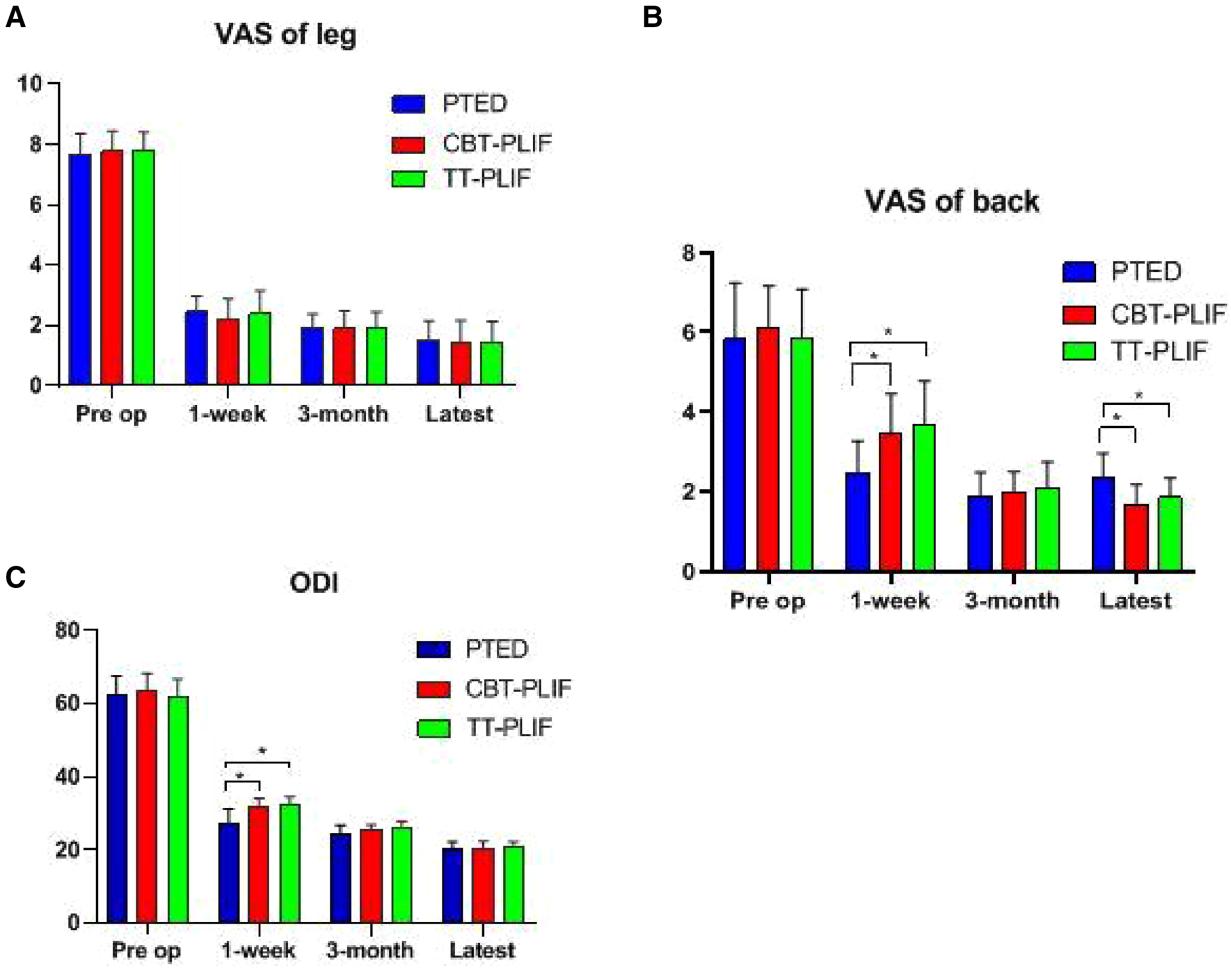
VAS and ODI improvements. Comparison of the (**A**) VAS score for leg pain, (**B**) VAS score for back pain, and (**C**) ODI at different time points.* *P* < 0.05, PTED group vs. CBT-PLIF group, PTED group vs. TT-PLIF group. VAS, visual analog scale; ODI, Oswestry Disability Index.

No significant differences were found among the three groups regarding the average VAS score for leg pain (*P > *0.05). The average VAS score for back pain in the PTED group was significantly reduced compared with those in the CBT-PLIF group and the TT-PLIF group at 1 week (*P *< 0.05); however, at the latest follow-up, the average VAS score for back pain in the CBT-PLIF group was significantly decreased compared with those in the other two groups (*P *< 0.05). The ODI scores in the PTED group were significantly reduced compared with those in the CBT-PLIF group and the TT-PLIF group at 1 week (*P *< 0.05). However, there was no significant difference among the three groups at the 3-month and the latest follow-ups regarding the ODI scores. Therefore, the CBT-PLIF group can significantly reduce back pain compared with the other two groups at the latest follow-up (Table 4, Figure 5).

The modified MacNab criteria were used to evaluate the global clinical outcomes. The good-to-excellent rate in the PTED group (82.35%) was reduced compared with those in the CBT-PLIF group (88.89%) and the TT-PLIF group (85.00%). However, there were no significant differences among the three groups regarding the good-to-excellent rate (Table 5).

**Table 5:** Comparison of modified MacNab evaluation criteria among three groups.

Groups	*n*	Excellent	Good	Fair	Poor
PTED	17	9	5	1	2
CBT-PLIF	9	5	3	1	0
TT-PLIF	20	13	4	3	0
*P*-value	0.557				

### 3.5. Comparison of complications and recurrence

Two complications were encountered (11.8%) in the PTED group, one (11.11%) in the CBT-PLIF group, and one (5%) in the TT-PLIF group. Two patients suffered numbness caused by irritation of exiting nerve roots following the procedure in the PTED group. Their neurological symptoms gradually diminished with the aid of rehabilitation and concomitant drugs. One patient in the CBT-PLIF group experienced CBT-PLIF screw dislocation, and then a revision surgery was performed to adjust the position of this screw. One patient in the TT-PLIF group experienced a small-sized dural tear (<5 mm). This participant complained of a headache, and his symptom improved following 1-week bed rest. There were no major complications such as neurovascular injury, cauda equina injury, surgical wound infection, and so on. There was no significant difference in the complication rates among the three groups (*P *> 0.05). Two patients experienced recurrent sciatica symptoms after a 6-week pain-free period in the PTED group. They were subjected to minimally invasive interbody fusion combined with CBT-PLIF screws fixation when conservative treatments had failed. Their neurological symptoms were alleviated up to the latest follow-up. Significant differences were found regarding the recurrence between PTED and the other two groups (*P *< 0.05) (Table 6).

**Table 6:** Comparison of complications and recurrence among three groups.

Groups	PTED	CBT-PLIF	TT-PLIF	*P*-value
Screw dislocation	0	1	0	0.030
Dysesthesia	2	0	0	
Dural tear	0	0	1	
Revision operation	2	0	0	

### 3.6. Representative cases

A 68-year-old man who underwent CBT-PLIF is presented in Figure 4.

## 4. Discussion

The present study has retrospectively compared three different techniques (PTED, CBT-PLIF, and TT-PLIF) for treating patients with symptomatic ASD following posterolateral lumbar fusion. Several studies have reported that PTED can be applied to treat symptomatic ASD with favorable clinical outcomes (15, 19). PTED has been widely accepted as an efficient treatment for symptomatic ASD. Therefore, PTED and conventional PLIF were applied as a reference to assess the efficiency and safety of CBT-PLIF. However, some studies have pointed out that the PTED approach can only achieve decompression of the nerve roots. The PTED approach cannot resolve the biomechanical effects caused by the adjacent fused segments. So, several radiological indicators were also used to evaluate the spine's stability following the revision surgery.

Posterolateral lumbar fusion combined with pedicle screw fixation (TLIF or PLIF) has been the most commonly used surgical procedure to treat lumbar degenerative diseases in the past 30 years. This method is efficient in managing LDH, lumbar stenosis, spondylolisthesis, and the like. However, it is combined with increased comorbidity, such as ASD. A prospective, randomized, long-term trial has demonstrated that fused lumbar segments significantly accelerate the occurrence of ASD compared with the natural course of lumbar disc degeneration. Moreover, long-segment fusion will further increase the occurrence of ASD compared with either short-segment fusion or other non-fusion approaches (20). The long-segment fusion might affect the global sagittal alignment of the lumbar spine and increase the ROM of adjacent segments compared with the short-segment fusion (21). This observation is consistent with the findings in our study; we also found that the occurrence of ASD is significantly increased in the long-segment fusion group compared with that in the short-segment fusion group (Table 1). In addition, similar to a previous study (22, 23), we also found that ASD superior to the fused segment is more often than ASD inferior to the fused segment in our study (Table 1). It is consistent with previous reports. We speculate that surgical manipulation might break the integrity of the adjacent joint capsule above the fused segments and further affect spinal stability in the future. It is more frequent in China that inexperienced residents usually perform the exposure procedure before internal fixation and decompression of nerve roots.

ASD is a specialized lumbar degenerative disease. Unlike the natural course of lumbar disc degeneration, ASD usually develops into symptomatic LDH within a relatively short period. Lumbar stenosis caused by hypertrophic ligament flavum or osteophyte of ASD is rare. In our study, lateral recess stenosis and foraminal stenosis caused by paramedian and far lateral LDH are two major types of pathologies. PTED with a transforaminal approach is preferred for managing lateral recess stenosis and foramen stenosis (24). Moreover, PTED is performed through a transforaminal approach, which can avoid the scar tissues caused by the previous operation (25). However, the technique applied in PTED for the management of symptomatic ASD is different from that used in conventional PTED. Due to blockage of spinal internal fixation, it is difficult to reach the target segment superior to the fused segments. It is the reason that more time is required for the treatment of symptomatic ASD superior to the fused segments via the transforaminal approach. The operator usually needs to increase the head tilt angle of the puncture needle to avoid blockage of internal spinal fixation. However, the puncture needle is closer to exiting nerve roots, and this manipulation increases the potential risk of exiting nerve root injury. Therefore, inferior foramina need to be expanded by foraminal-plasty under endoscopy to reduce the occurrence of exiting nerve roots injury.

CBT screw is an alternative solution to the pedicle screw in posterior spinal instrumentation (26). With the generalization and popularization of the CBT screw application, CBT-PLIF offers an alternative approach for managing symptomatic ASD. Several studies have demonstrated that CBT-PLIF can achieve favorable clinical outcomes in treating symptomatic ASD (27–29). It can offer several advantages compared to TT-PLIF. CBT screws can increase the contact area between the screw and cortical bone and enhance the biomechanical stability of fixation. Biomechanical tests have demonstrated that CBT screws can improve pullout strength compared to a pedicle screw fixation system. Moreover, CBT-PLIF can significantly reduce the damage to paraspinal muscle and preserve the integrity of the facet joint capsule compared with conventional TLIF or PLIF operation. Several studies have reported that CBT-PLIF can significantly reduce back pain caused by iatrogenic muscle injury and improve functional recovery compared with TT-PLIF (26, 30).

Although PTED can significantly reduce the operation time, incision length, intraoperative blood loss, and time to return to work compared with CBT-PLIF, dynamic instability factors following PTED still exist, and fused segments will still increase the ROM of adjacent segments. Two patients in our study experienced a recurrence of LDH following PTED. A previous study has reported similar findings that patients with symptomatic ASD underwent revision surgery following PTED due to biomechanical interruption of the lumbosacral spine (7). Unlike PTED, the other minimally invasive surgery applied in this study, CBT-PLIF can provide superior biomechanical stability to the lumbosacral spine following decompression. Two patients experienced a recurrence of LDH following PTED, and no one experienced it in the CBT-PLIF group. We speculate that lumbar fusion combined with CBT screws can re-establish the abnormal sagittal balance of the lumbosacral spine that might result in aggressive changes in ROM following revision surgery.

In our study, the postoperative VAS score for back pain was significantly decreased in the PTED group compared with the those in CBT-PLIF group and the TT-PLIF group at 1-week follow-ups. This is because the PTED procedure can significantly reduce the exposure of the paraspinal muscle compared with the CBT-PLIF group and the TT-PLIF group. Several studies have demonstrated that paraspinal musculature resulting from denervation and ischemia is closely related to postoperative back pain within a short period (7, 8, 15). However, at the latest follow-up, the average VAS score for back pain in the CBT-PLIF group was significantly decreased compared with those in the TT-PLIF group and the PTED group. We speculated that the paraspinal muscle caused by CBT-PLIF recovered from injury and kept normal mechanical properties in the long term. Biomechanical studies have demonstrated that spinal instability is associated with low back pain. No obvious difference was found in VAS score for leg pain among the three groups. It indicates that three different approaches can achieve satisfactory outcomes in relieving symptoms of radiating pain. The VAS score for leg pain is closely related to the decompression procedure. The ODI score has also been applied to evaluate functional recovery. A previous study has reported that a 15% improvement in the ODI score can be defined as favorable surgical outcomes. The data in our study were consistent with these criteria. However, the ODI score was significantly reduced in the CBT-PLIF group and the TT-PLIF group compared with that in the PTED group at 1-week follow-ups. The ODI score is similar to the changes in back pain VAS score for back pain. Some studies have shown that the ODI score was consistent with the VAS score (31). The good-to-excellent outcome rates were 82.35%, 88.89%, and 85.00%, respectively, similar to previous reports (32, 33).

Postoperative dysesthesia is one of the most common morbidities following endoscopic surgery (34, 35). Two patients in the PTED group experienced postoperative dysesthesia (11.8%), which was higher than previous reports [4.89% (0%–9.76%)] (7, 33). We attribute this to the following reasons. First, as mentioned before, blockage of internal spinal fixation is technically challenging for surgeons, and increased puncture attempts by error and trail might irritate exiting nerve roots. One case with a dural matter tear was observed in the TT-PLIF group. TT-PLIF was applied to treat symptomatic ASD in the case series earlier. It is a more extensive procedure compared with PTED and CBT-PLIF. The operative scar caused by previous surgery might increase the chance of a dural matter tear. One patient had one screw malpositioned in the TT-PLIF group; navigation-guided implantation could be able to avoid this and is recommended (36). Two patients (11.8%) suffered from a recurrence in the PTED group. We speculated that it is due to the postoperative spinal instability caused by biomechanical interruption of the lumbosacral spine; however, a large-scale and long-term study should be conducted to verify this hypothesis.

The main limitations of our study are the relatively small sample size and retrospective nature. Because this was a retrospective study, all baseline features could not be strictly controlled for, and there was a selection bias for patients. Larger cohort studies or randomized controlled studies are necessary to confirm our results, and the next step is to demonstrate that CBT-PLIF has better biomechanical stability than TT-PLIF. Another major limitation is the choice of surgical approaches. In this study, the choice of surgical procedure is determined by the surgeon based on the requirements of patients and the surgical expertise of the operators. Despite these limitations, this study is of great value because it is the first to compare the three techniques in treating adjacent segment degeneration after lumbar fusion.

## 5. Conclusions

All three approaches can treat symptomatic ASD efficiently and effectively. Functional recovery was more accelerated in the PTED group compared with the other approaches in the short term. However, CBT-PLIF and TT-PLIF can provide superior biomechanical stability to the lumbosacral spine following decompression compared with PTED, and compared with TT-PLIF, CBT-PLIF can significantly reduce back pain caused by iatrogenic muscle injury and improve functional recovery. Therefore, superior clinical outcomes were achieved in the CBT-PLIF group compared with the PTED and TT-PLIF groups in the long term.

## Data Availability

The original contributions presented in the study are included in the article/Supplementary Material; further inquiries can be directed to the corresponding author.
